# 1,2-Dichlorobenzene affects the formation of the phosphoenzyme stage during the catalytic cycle of the Ca^2+^-ATPase from sarcoplasmic reticulum

**DOI:** 10.1186/s12858-016-0061-1

**Published:** 2016-03-11

**Authors:** Javier Vargas-Medrano, Jorge A. Sierra-Fonseca, Luis F. Plenge-Tellechea

**Affiliations:** Departamento de Ciencias Químico Biológicas, Laboratorio de Biología Molecular y Bioquímica (Edif. T-216), Instituto de Ciencias Biomédicas, Universidad Autónoma de Ciudad Juárez, Plutarco Elías Calles #1210 Fovissste Chamizal, Ciudad Juárez, Chihuahua C.P. 32310 Mexico; Present address: Department of Biological Sciences, University of Texas at El Paso, El Paso, TX 79968 USA; Present address: Department of Biomedical Sciences, Center of Emphasis for Neurosciences, Texas Tech University Health Science Center, El Paso, TX 79905 USA

**Keywords:** Ca^2+^-ATPase, Sarcoplasmic reticulum, Dichlorobenzene, Phosphoenzyme

## Abstract

**Background:**

1,2-Dichlorobenzene (1,2-DCB) is a benzene-derived molecule with two Cl atoms that is commonly utilized in the synthesis of pesticides. 1,2-DCB can be absorbed by living creatures and its effects on naturally-occurring enzymatic systems, including the effects on Ca^2+^-ATPases, have been poorly studied. Therefore, we aimed to study the effect of 1,2-DCB on the Ca^2+^-ATPase from sarcoplasmic reticulum (SERCA), a critical regulator of intracellular Ca^2+^ concentration.

**Results:**

Concentrations of 0.05–0.2 mM of 1,2-DCB were able to stimulate the hydrolytic activity of SERCA in a medium-containing Ca^2+^-ionophore. At higher concentrations (0.25–0.75 mM), 1,2-DCB inhibited the ATP hydrolysis to ~80 %. Moreover, ATP hydrolysis and Ca^2+^ uptake in a medium supported by K-oxalate showed that starting at 0.05 mM,1,2-DCB was able to uncouple the ratio of hydrolysis/Ca^2+^ transported. The effect of this compound on the integrity of the SR membrane loaded with Ca^2+^ remained unaffected. Finally, the analysis of phosphorylation of SERCA by [γ-^32^P]ATP, starting under different conditions at 0° or 25 °C showed a reduction in the phosphoenzyme levels by 1,2-DCB, mostly at 0 °C.

**Conclusions:**

The temperature-dependent decreased levels of phosphoenzyme by 1,2-DCB could be due to the acceleration of the dephosphorylation mechanism – E_2_P · Ca_2_ state to E_2_ and P_i_, which explains the uncoupling of the ATP hydrolysis from the Ca^2+^ transport.

## Background

1,2-Dichlorobenzene (1,2-DCB) is a chlorinated benzene molecule used as precursor in the synthesis of pesticides. 1,2-DCB can be absorbed and accumulated by humans and wildlife, and it has been detected in different biological fluids. Moreover, 1,2-DCB has been linked to severe human health problems such as liver damage and anemia [1–6]. Oral administration of a radioactively-labeled 1,2-DCB to rats revealed that it can be distributed among all tissues, including skeletal muscle. In addition, it has been shown that DCB molecules can induce a rise in intracellular Ca^2+^ in human neuronal SH-SY5Y cells [7, 8]. Given this evidence, we hypothesized that 1,2-DCB can affect the functionality of skeletal muscle proteins, particularly the Ca^2+^-ATPase from sarcoplasmic reticulum (SERCA), a predominant protein of skeletal muscle, a hypothesis that has not previously been explored. SERCA is a large (110-kDa) sarcoplasmic reticulum protein with 10 transmembrane regions, and it plays the essential role of reducing the intracellular Ca^2+^ concentration by consuming ATP as a source of energy [9–12]. Each molecule of SERCA protein utilizes the energy of one ATP molecule to pump 2Ca^2+^ across the sarcoplasmic membrane, thus maintaining low cytoplasmic Ca^2+^ concentrations and high intravesicular Ca^2+^ concentrations [13, 14]. The catalytic cycle of SERCA involves two main conformational states, E_1_ with high-Ca^2+^ and E_2_ with low-Ca^2+^ affinities [15–17]. In addition, the three-dimensional structures of the two conformational stages of SERCA and phosphorylated intermediate (EP) have been successfully determined [18–22]. Furthermore, the coupling ratio of both transported Ca^2+^ and hydrolyzed ATP occurs through a sequence of phosphorylated and non-phosphorylated enzymatic intermediates, with or without Ca^2+^ bound to the enzyme, and it has been shown that EP accumulation can serve as an indicator of enzyme turnover, which can be affected by a variety of factors such as temperature, nature of the substrate, Ca^2+^ concentration and presence of organic solvents [23].

Given the critical role of SERCA in maintaining appropriate cytosolic Ca^2+^ levels, chemical agents that affect its functionality can have harmful effects to the overall cellular function. In this regard, a multitude of molecules have been described to act as inhibitors of SERCA [24]. Naturally-occurring compounds such as cyclopiazonic acid (a fungal toxin), and thapsigargin (a sesquiterpene lactone) are two of the most widely used inhibitors of SERCA [25–27]. Inorganic compounds such as vanadate, tungstate and molybdate have also been reported to inhibit SERCA activity [28–31]. Other natural compounds such as ellagic acid and gingerol, which have been described to stimulate SERCA activity, have been shown to be promising therapeutic targets for cardiovascular dysfunction [32, 33]. In addition, several molecules, including thapsigargin derivatives and flavonoids are being developed as potential cancer treatments based on their SERCA-mediated anti-proliferative and pro-apoptotic effects [34, 35]. Furthermore, a broad variety of organic solvents and hydrophobic compounds bearing nucleophilic groups have been extensively reported as inhibitors of SERCA activity [36–41]. The inhibitory effect of these compounds was related to their hydrophobicity, and notably, they were also able to stimulate SERCA activity at certain concentrations. In this study, we provide evidence, for the first time, that 1,2-DCB affects Ca^2+^ homeostasis by affecting SERCA, a fundamental Ca^2+^ modulator. We report here the effect of 1,2-DCB on several key features of SERCA, including ATP hydrolytic activity, the ratio of hydrolysis of ATP/Ca^2+^ transported, and the phosphorylation stage (EP) during the catalytic cycle of this enzyme.

## Methods

### Chemicals and reagents

[γ-^32^P]ATP and ^45^CaCl_2_ were purchased from Perkin-Elmer, USA. [^3^H]Glucose and ^45^CaCl_2_ were products of DuPont NEN. The Ca^2+^ ionophore A23187 (calcimycin), EGTA (ethylene glycol-bis (β-aminoethyl ether)-N,N,N’,N’-tetra acetic acid), Mops (4-morpholinepropanesulfonic acid), liquid scintillation cocktail (Sigma-Fluor S-4023), ATP disodium salt, LaCl_3_, K-oxalate, benzene, 1,2-DCB was obtained from Sigma, Co. (St. Louis, MO). CaCl_2_ and other chemicals ware purchased from J.T. Baker (México). The Ca^2+^ ionophore A23187 was dissolved in ethanol. Benzene and 1,2-DCB were dissolved in methanol. Ethanol and methanol never reached a concentration higher than 1 % (v/v) in the reaction media after the addition of 1,2-DCB or A23187.

### Preparation of sarcoplasmic vesicles

SR (sarcoplasmic reticulum) membranes rich in Ca^2+^-ATPase (SERCA) were prepared from the low density SR of skeletal fast-twitch muscle of adult New Zealand rabbit hind limbs, and preserved in a buffer containing 10 mM Mops, pH 7.0 and 30 % sucrose and stored at −80 °C as described by Eletr and Inesi [42]. The sarcoplasmic membrane concentration refers to milligrams (mg) of total protein per milliliters (ml) and was measured by the colorimetric procedure described by Lowry et al. [43]. We used bovine serum albumin as standard protein.

### Free Ca^2+^ concentration in the reaction media

Free Ca^2+^ concentration was adjusted by adding the appropriate volume of CaCl_2_ and EGTA to the enzymatic reaction media [44]. Free Ca^2+^ concentration was calculated by the computer program *Calcium* which is based on the absolute constant stability for the Ca^2+^-EGTA complex, the EGTA protonation equilibrium, Ca^2+^ ligands and media pH [45, 46].

### Ca^2+^-ATPase (SERCA) hydrolytic activity supported by A23187 or K-oxalate

Initial rates of ATP hydrolysis by SR vesicles were measured at 25 °C for 5 min (min) by following the liberation of inorganic phosphate (P_i_). A typical assay medium was buffered at pH 7.0, and contained 20 mM Mops, pH 7.0, 80 mM KCl, 5 mM MgCl_2_, 1 mM EGTA, 0.967 mM CaCl_2_ (10 μM free Ca^2+^),0.01 mg/ml SR vesicles, 1.5 μM A23187 (to generate leaky vesicles to measure in min) or 5 mM K-oxalate (no leaks and to measure in min). A typical reaction was started after the addition of 1 mM of ATP. Any change in the concentration of SR protein added to the reaction medium is detailed in the figure captions. The compound 1,2-DCB was added at the indicated concentrations (0.05–1 mM). The appearance of P_i_ from the hydrolysis of ATP mediated by SERCA was evaluated with a molybdovanadate reagent previously described by Lin and Morales [47]. Experimental details are indicated in the corresponding figure legends.

### Ca^2+^ uptake supported by K-oxalate

SERCA is a Ca^2+^ pump that depends on Mg^2+^ and the presence of ATP to transport Ca^2+^ across of the SR membrane. For this reason, we studied the Ca^2+^ transported by SERCA in the presence of 1,2-DCB. Ca^2+^ transported by SERCA was measured at 25 °C using ^45^CaCl_2_ as a radioactive tracer followed by sample filtration according with the methods described by Martonosi and Feretos [48]. The experiment was conducted in a reaction mixture containing 20 mM Mops, pH 7.0, 80 mM KCl, 5 mM MgCl_2_, 1 mM EGTA, 5 mM K-oxalate instead of ionophore A23187 (to keep vesicles intact), 0.967 mM CaCl_2_ (10 μM free Ca^2+^) with 1000 cpm ^45^CaCl_2_ per nmol of Ca^2+^, and different concentrations of 1,2-DCB. The reaction contained 0.01 mg/ml of protein and was started after adding 1 mM of ATP. Ca^2+^ uptake mediated by SERCA was stopped by filtering the vesicles in 0.45 μm HA type nitrocellulose membranes filters (Millipore, Milford, MA). The filters containing the Ca^2+^-loaded SR vesicles were washed two times with 2 ml of a buffer containing 20 mM Mops pH 7.0, 80 mM KCl, 5 mM MgCl_2_ and 1 mM LaCl_3_. Finally, ^45^Ca^2+^ transported was measured by scintillation spectroscopy using 3 ml of scintillation cocktail per vial. The use of radioactive standards allowed us to express the Ca^2+^ uptake data as nmol Ca^2+^/mg of protein.

### Membrane integrity test

In order to determine if 1,2-DCB was affecting the membrane integrity and therefore interfering with Ca^2+^ measurements, SR vesicles (0.01 mg/ml of protein) were loaded with ^45^Ca^2+^ by performing a Ca^2+^ transport assay using the active transport of SERCA as described before for Ca^2+^ uptake [48]. The reaction was started with 1 mM of ATP and the reaction ran for 5 s (s) until SR vesicles were loaded with detectable amounts of Ca^2+^. In order to determine if DCB induced vesicle leakage, the ^45^Ca^2+^-loaded vesicles were exposed to 1 mM of a 1,2-DCB right before stopping the reaction by filtration. In addition, two positive controls were incorporated in substitution for oxalate: Ca^2+^ ionophore (1.5 μM of A23187) in combination with 1 mM 1,2-DCB, and Ca^2+^ ionophore alone. Very importantly, A23187 does not allow Ca^2+^ to be accumulated into SR vesicles. For more details of the experiment please review Ca^2+^ uptake supported by oxalate above. The amount of ^45^Ca^2+^ loaded into the vesicles was determined by scintillation spectroscopy.

### Ca^2+^ binding at equilibrium assay

The binding of Ca^2+^ to SERCA was tested in the absence of ATP using ^45^CaCl_2_. Unbound Ca^2+^ was evaluated from the filter wet volume by using [^3^H]glucose as a marker. This approach was used to test the Ca^2+^ high-affinity conformational state of SERCA (E_1_Ca_2_ stage). The effect of 1,2-DCB on E_1_Ca_2_ state was evaluated at 25 °C for 5 min in a Ca^2+^-saturating medium containing 20 mM Mops, pH 7.0, 5 mM MgCl_2_, 80 mM KCl, 0.1 mM EGTA, 0.105 mM [^45^Ca]CaCl_2_ (~5000 cpm/nmol) (10 μM free Ca^2+^), 1 mM [^3^H]glucose (~10,000 cpm/nmol), and 0.2 mg/ml of SR protein. Subsequently, we added different concentrations of 1,2-DCB. In order to test the effect of 1,2-DCB on E_2_ stage we used a Ca^2+^-free media containing 20 mM Mops, pH 7.0, 5 mM MgCl_2_, 80 mM KCl, 0.1 mM EGTA, 1 mM [^3^H]glucose (~10,000 cpm/nmol), and 0.2 mg/ml of SR protein, and different concentrations of 1,2-DCB. The incubation was performed at 25 °C for 5 min. Later, the reaction for E_2_ stage was supplemented by adding a buffer containing 20 mM MOPS, pH 7.0, 5 mM MgCl_2_, 80 mM KCl, 0.1 mM EGTA, 3.15 mM [^45^Ca]CaCl_2_ (~5000 cpm/nmol) and 1 mM [^3^H]glucose (~10,000 cpm/nmol) with a final free-Ca^2+^ concentration of 10 μM. Following, the assay was prolonged for 1 min at 25 °C. Samples of 1 ml (0.2 mg of protein) were filtered under vacuum in 0.45 μm nitrocellulose filters (Millipore, HA type). Any further washing to determine ^45^Ca and ^3^H associated with the filters and the amount of radioactivity in the filters was determined by scintillation spectroscopy. Specific binding of Ca^2+^ to the SERCA was calculated by subtracting unbound Ca^2+^ retained by the filter.

### Phosphorylation of SERCA by [γ^32^]ATP

Maximal levels of EP (phosphorylated enzyme intermediate) were determined using [γ-^32^P]ATP as a substrate and starting from different incubation conditions: E_1_Ca_2_ -DCB, E_2_ -DCB and E_2_[γ-^32^P]ATP-DCB in similar manner as we described in previous work [25, 26]. These conditions were studied during the enzyme turnover by mixing equal volumes (0.5 ml) of SR vesicles with the phosphorylating medium with or without Ca^2+^ (after both solutions were mixed, the final volume was 1 ml and 0.1 mg/ml of SR protein). The SR vesicles (0.2 mg protein/ml) were suspended in a tube with 0.5 ml of 20 mM Mops, pH 7.0, 80 mM KCl, 5 mM MgCl_2_, 1 mM EGTA, 0.967 mM CaCl_2_ (10 μM free Ca^2+^), and 15 μM A23187. To measure E_1_Ca_2_-DCB stage, the reaction mixture was preincubated with a defined concentration of 1,2-DCB and the phosphorylation reaction was started by mixing 0.5 ml of this medium with 20 μl of medium containing 20 mM Mops, pH 7.0, 80 mM KCl, 5 mM MgCl_2_, 1 mM EGTA, 0.967 mM CaCl_2_, and 1.25 mM [γ-^32^P]ATP (50 μM final concentration, ~20,000 cpm/nmol). When the enzyme (0.2 mg/ml) was in nominally Ca^2+^-free medium (E_2_ -DCB stage), the phosphorylating medium contained CaCl_2_ to give 10 μM free Ca^2+^ after mixing. In order to determine if 1,2-DCB was directly affecting the E_2_ATP in a Ca^2+^ free medium, the formation of the EP stage was started when Ca^2+^ (to give 10 μM free) was added into the reaction medium. Phosphorylation time was 2 s when the enzyme turnover was studied at 0 °C, and 5 s when studied at 25 °C. The reaction was started under continuous vortexing and stopped by adding 1 ml of ice-cold 250 mM perchloric acid and 2 mM sodium phosphate. Stopped reactions were incubated on ice for 5 min before filtration through 0.45 μm nitrocellulose filters (Millipore, HA type). SR vesicles retained by the filters were washed 5 times with ice-cold 250 mM perchloric acid and 2 mM sodium phosphate. Finally, the amount of radioactivity in the filters was determined by scintillation spectroscopy. These results are expressed as nmol EP/milligram of protein.

### Statistical analysis

Statistical and kinetic analysis was performed using Sigma Plot 11 software. Unless otherwise stated, the experiment values are represented as the mean of at least three independent experiments, each performed in triplicate +/− average SE, with statistical significance of *p* < 0.05 determined by t-student’s paired *t*-test.

## Results

### Effect of 1,2-DCB on the hydrolytic activity of SERCA supported by A23187

**Fig. 1:**
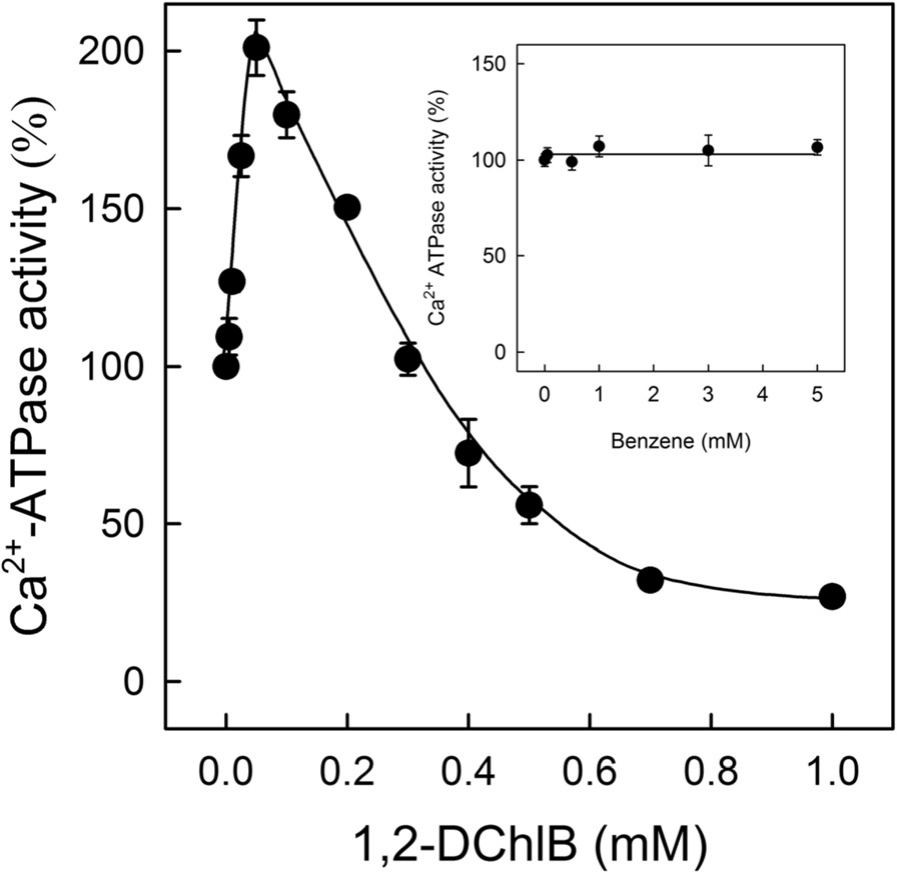
Effect of 1,2-DCB and benzene on the initial rates of ATP hydrolysis supported by A23187. The reaction was performed in a buffer containing 20 mM Mops, pH 7.0, 80 mM KCl, 5 mM MgCl2, 1 mM EGTA, 0.967 mM CaCl2, and 1.5 μM A23187. The reaction was conducted by adding 0.01 mg/ml of SR vesicles with different concentrations of 1,2-DCB (mM) or benzene (mM). First data point corresponds to Ca^2+^-ATPase activity without DCB (control) and it was ~2.6 ± 0.05 μmol P_i_/mg∙min

In order to determine the effect of 1,2-DCB on the ATP hydrolytic activity of SERCA, we titrated different 1,2-DCB concentrations in a typical Ca^2+^-ATPase assay where SERCA hydrolyzed ATP to ADP and P_i_ as described before. In this experiment we incubated SR vesicles with A23187, a Ca^2+^ ionophore, which is used to generate leaky vesicles (absence of gradient) in order to avoid saturation of SR vesicles with Ca^2+^, which would otherwise stop Ca^2+^-ATPase activity. The dose-response curve revealed a biphasic effect of 1,2-DCB on the ATP hydrolytic activity of SERCA: while lower concentrations of the compound highly stimulated ATP hydrolysis, 1,2-DCB concentrations higher than 0.3 mM inhibited SERCA hydrolytic activity (Fig. 1). In addition, 0.5 mM of 1,2-DCB reduced SERCA activity to 50 % (IC_50_ = 0.5 mM). To determine the importance of the Cl atoms on the effect of the compound on SERCA hydrolytic activity, we used the benzene molecule, which is the same ring base structure, but depleted of Cl atoms (Fig. 1, inset). A dose-response curve was performed by titrating several benzene concentrations, and we found that the benzene molecule by itself did not affect SERCA hydrolytic activity at all, indicating that Cl atoms are involved in the biphasic effect observed on SERCA hydrolytic activity.

### Effect of 1,2-DCB on Ca^2+^ uptake and ATP hydrolysis ratio mediated by SERCA and supported by K-oxalate

**Fig. 2:**
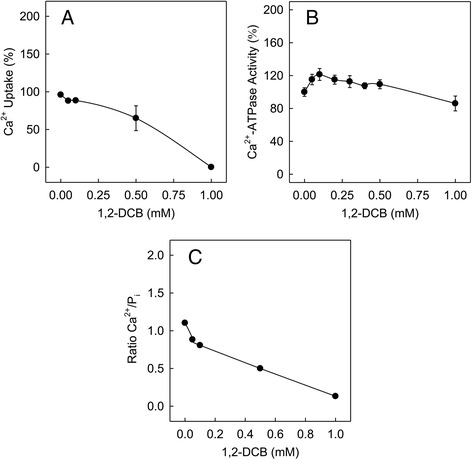
The Ca^2+^-ATPase activity supported by oxalate was uncoupled by 1,2-DCB. The effect of 1,2-DCB on Ca^2+^/P_i_ coupling ratio was determined. The coupling ratio at each DCB concentration was calculated as the ratio between the Ca^2+^ uptake rate and the ATP hydrolysis rate by SERCA. SR vesicles (0.01 mg/ml) were equilibrated at 25 °C in a medium containing 5 mM K-oxalate. Hydrolysis rate of ATP (**a**) or ^45^Ca^2+^ uptake rates (**b**) were measured at same experimental conditions. **c** The Ca^2+^/P_i_ coupling ratio (calculated by dividing the Ca^2+^ transport values by those of ATP hydrolysis), in presence of different concentrations of 1,2-DCB was uncoupled

We next aimed to determine the effect of 1,2-DCB on Ca^2+^ uptake mediated by SERCA. Vesicular fragments of SR exhibit linear rates of Ca^2+^ transport upon addition of ATP when equilibrated in the presence of K-oxalate as a lumenal Ca^2+^ regulator (non leaky vesicles). In order to avoid Ca^2+^ leakage caused by the Ca^2+^ ionophore (A23187), we used K-oxalate in both Ca^2+^-transport and hydrolytic activity experiments in order to make them comparable to Ca^2+^ transport. The dose-response curves showed that 1,2-DCB did not stimulate Ca^2+^ uptake mediated by SERCA, but in contrast, Ca^2+^ uptake was inhibited by DCB concentrations above 0.5 mM until achieving full inhibition at 1 mM (Fig. 2a). Ca^2+^ transport cannot be measured in an assay containing a Ca^2+^ ionophore (as used to measure the ATP hydrolysis) therefore these experimental conditions did not allow us to compare the Ca^2+^ uptake assay with the previously conducted experiments of hydrolytic activity. The approach employed for Ca^2+^ uptake measurement is less sensitive than the one using Ca^2+^ ionophore and reduces enzyme activity by ~1 fold, thus showing less effect of 1,2-DCB (Fig. 2b), however, there were peaks of activation of approximately 15 and 20 % at DCB concentrations of 0.05 and 0.1 mM, respectively. Nevertheless, when we compared the ATP hydrolysis results with the ones conducted for Ca^2+^ uptake, we were able to find a disruption in the Ca^2+^ uptake/ATP hydrolysis ratio (calculated by dividing the Ca^2+^ transport values by those of ATP hydrolysis), which indicates that the enzyme activity ratio was uncoupled, as opposed to normal physiological conditions during which the enzyme maintains a coupled ratio of 2Ca^2+^ for each mol of ATP hydrolyzed to ADP and P_i_ (Fig. 2c). The observed ratio appears to confirm that 1,2-DCB concentrations greater than 0.05 mM cause a disturbance on the enzyme activity, an effect that is not observed only with the hydrolysis.

**Fig. 3:**
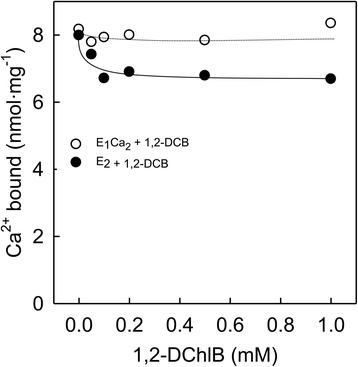
Binding of Ca^2+^ to SERCA. To test if 1,2-DCB was affecting the binding of Ca^2+^ to the enzyme we performed a binding assay as described in *Methods*. The complex Ca^2+^/SERCA (E_1_Ca_2_) (o) or enzyme prior binding to Ca^2+^ (E_2_) (●) were exposed to different concentrations of 1,2-DCB

Given that 1,2-DCB affected Ca^2+^ uptake mediated by SERCA, we aimed to determine if 1,2-DCB was also affecting the binding of Ca^2+^ to SERCA. Direct measurements of ^45^Ca^2+^ binding to the non-phosphorylated enzyme pre-incubated with the compound indicated that the E_1_ conformation remained unaffected by treatment with 1,2-DCB (Fig. 3, open circles). In contrast with the E_1_ form, treatment with 1,2-DCB starting from the E_2_ form prior to binding of Ca^2+^ affected only ~16 % of the total Ca^2+^ bound to the enzyme (Fig. 3, closed circles), but the levels remained constant with all 1,2-DCB concentrations.

### Membrane protein-dependent effect of 1,2-DCB and SR membrane integrity

**Fig. 4:**
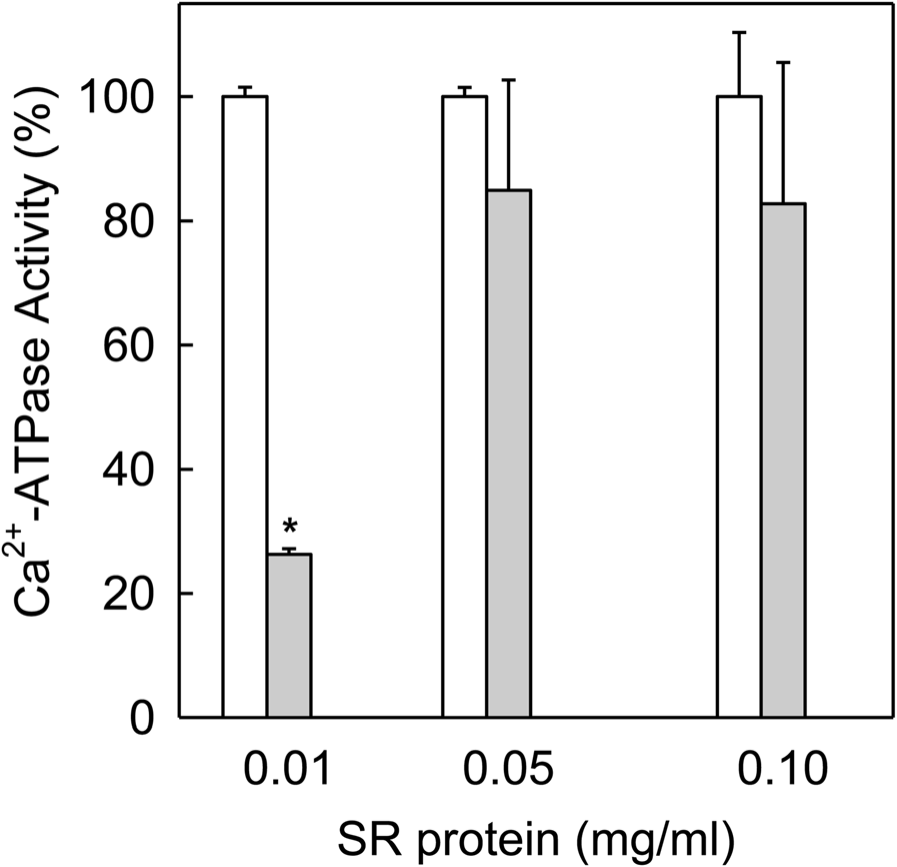
The effect of 1,2-DCB was SR vesicles-dependent. The assay medium contained 20 mM Mops, pH 7.0, 80 mM KCl, 5 mM MgCl_2_, 1 mM EGTA, 0.967 mM CaCl_2_, different SR protein concentrations (0.01 and 0.10 mg/ml), and 1.5–15 μM A23187 (A23187 was proportionally increased with respect SR total protein). SERCA activity was assayed at 25 °C in absence of compound as control (white bars) and with 1 mM 1,2-DCB (gray bars). The reactions started by the addition of 1 mM ATP. Controls were normalized to 100 % of Ca^2+^-ATPase activity and the actual values were 2.6 ± 0.05, 2 ± 0.04, and 1.6 ± 1.3 μM P_i_/min/mg. *, *p* = <0.001, compared to their respectively control that does not have DCB

**Fig. 5:**
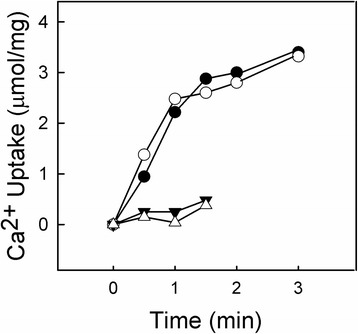
Time-dependent filling of SR vesicles and exposure to 1,2-DCB. In order to determine the effect of 1,2-DCB on the filling of the vesicles by SERCA protein, we exposed on the SR membrane, ^45^Ca^2+^ uptake was measured under same conditions described for Ca^2+^ transport. At different times in Ca^2+^ uptake, 1,2-DCB (open circles) was added to the reaction media before stopping the reaction. The same reaction without DCB treatment was tested as a control (closed circles). Two additional positive controls were incorporated in substitution for oxalate, Ca^2+^ ionophore (1.5 μM of A23187) was added to the assays with (closed triangles) or without 1 mM 1,2-DCB (open triangles). Very importantly, A23187 did not allow Ca^2+^ to be accumulated into SR vesicles

1,2-DCB is a typical hydrophobic molecule and as such, it has affinity for hydrophobic environments such as biological membranes. For this reason, we used our model of study to investigate the possibility of 1,2-DCB targeting the SR membranes. First, we conducted a set of experiments using a high-dose of 1,2-DCB in reactions having 10-fold different amounts of SR vesicles (0.01–0.1 mg/ml total protein). In these experiments we tested hydrolysis of ATP mediated by SERCA and we found that the inhibitory effect of 1,2-DCB was significantly reduced with high amounts (5–10 folds higher) of SR vesicles (0.05–0.1 mg/ml total membrane protein), which strongly suggested that 1,2-DCB molecules were being diluted into the SR membranes (Fig. 4). These results indicate that DCB molecules were going from the aqueous environment of the reaction medium into the membranes, and when the number of SR vesicles was increased, there were less 1,2-DCB molecules per SR membrane. However, this does not confirm if 1,2-DCB was affecting the integrity of the SR membrane. In order to analyze if 1,2-DCB molecules were disrupting the integrity of the SR membrane, we loaded SR vesicles with Ca^2+^, using a typical Ca^2+^ uptake assay as previously described, and 5 s before stopping the reaction we added 1 mM of 1,2-DCB into the reaction mix (Fig. 5). SR vesicles treated with 1,2-DCB (open circles) did not show any difference in levels of accumulated Ca^2+^ inside of the SR vesicle when compared to controls (without 1,2-DCB, closed circles), thus demonstrating that 1,2-DCB did not disrupt membrane vesicles. In addition, we performed a positive control with a Ca^2+^ ionophore (A23187) knowing that it produces leaky vesicles and therefore does not allow Ca^2+^ accumulation (Fig. 5, closed and open triangles). As observed, vesicles with A23187 were only able to accumulate ~11 % of the total Ca^2+^ accumulated in the control at 1 min of filling (100 %). This demonstrates that, at least in short time exposure, DCB does not disrupt the SR membrane integrity, which indicates that the effect of 1,2-DCB on SERCA hydrolytic activity was due to an effect on SERCA functionality.

### Formation of EP stage during the catalytic cycle of SERCA was inhibited by 1,2-DCB

**Fig. 6:**
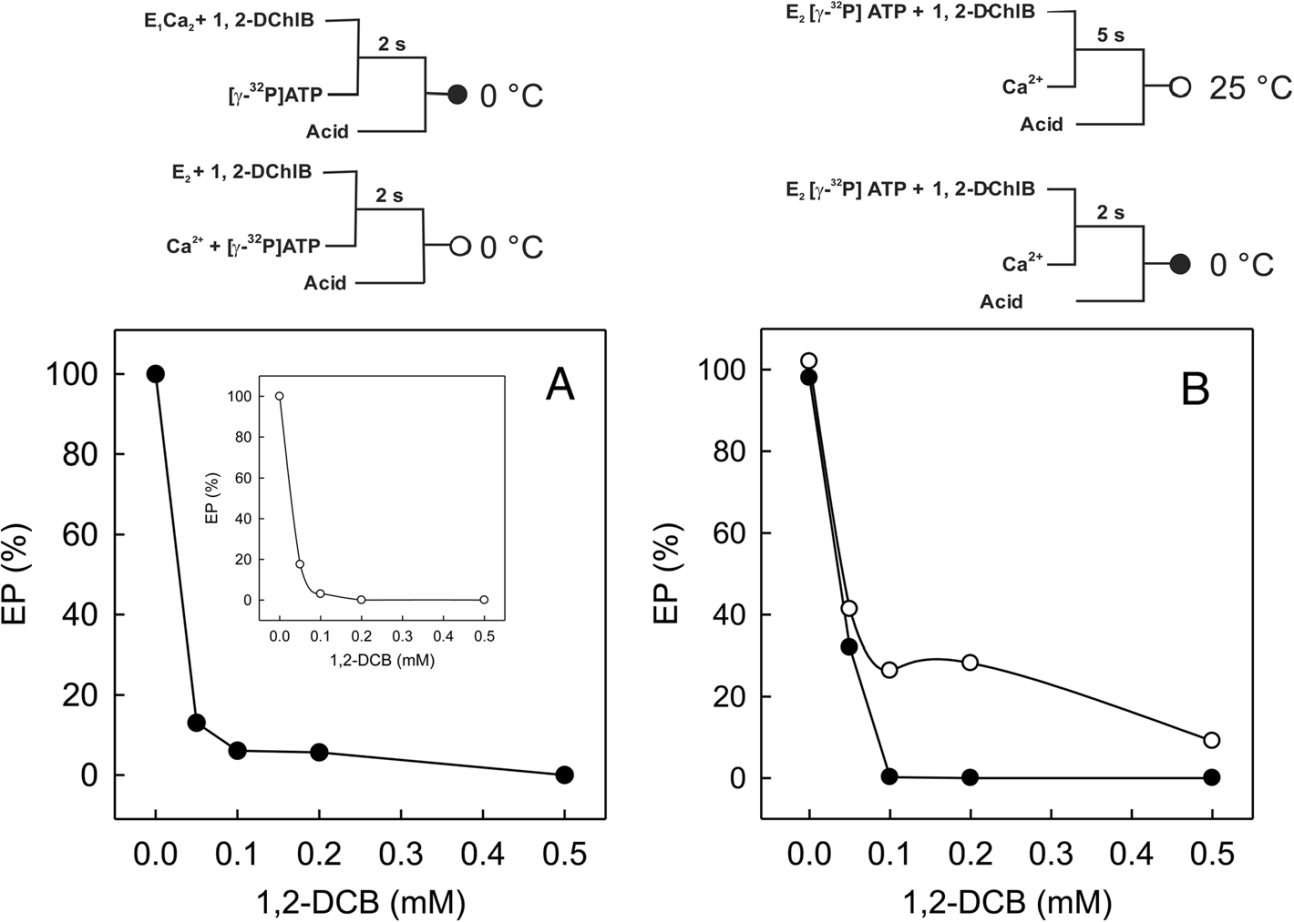
Effect of 1,2-DCB on functional states of SERCA as determined by ATP phosphorylation. SR vesicles at 0.1 mg of protein/ml were incubated at 25 °C in a medium containing: **a** 20 mM Mops, pH 7.0, 80 mM KCl, 5 mM MgCl_2_, 15 μM A23187, 0.1 mM EGTA, 10 μM free Ca^2+^, and a certain 1,2-DCB concentration starting with 50 μM [γ-^32^P]ATP (●). *Inset:* The E_2_ + 1,2-DCB prior phosphorylation was starting with 10 μM free Ca^2+^ and 50 μM [γ-^32^P]ATP (○). The phosphorylation in each case was maintained for 2 s at 0 °C. **b** For phosphorylation initiating from E_2_ATP Ca^2+^ free, the enzyme (0.1 mg of protein/ml) was first treated with 1,2-DCB in a media containing 20 mM Mops, pH 7.0, 80 mM KCl, 5 mM MgCl_2_, 15 μM A23187, 0.1 mM EGTA, followed by phosphorylation at 0 °C or 25 °C by adding [γ-^32^P]ATP and 10 μM free Ca^2+^. For more details see *Methods*

The following step was to study the formation of EP stage by ATP during the turnover of SERCA, which is a crucial step in the catalytic cycle of this enzyme. Surprisingly, all the 1,2-DCB concentrations tested considerably reduced the formation of the E_1_P stage of SERCA (Fig. 6a). When starting from E_1_Ca_2_ plus 1,2-DCB, and the variation E_2_ plus 1,2-DCB started with Ca^2+^/ATP (inset), the EP levels were drastically reduced: E_1_Ca_2_ with 0.05 mM of 1,2-DCB barely reached 13.04 % of accumulated EP, with 0.1 and 0.2 mM of 1,2-DCB further reducing EP levels to 6.08 and 5.65 %, respectively and 0.5 mM of the compound completely abolishing EP accumulation. In the case of E_2_, the EP levels were similarly affected: 0.05 mM of 1,2-DCB decreased EP to 17.5 %, with 0.1 mM further reducing it to 3.0 %, and the remaining concentrations showed no EP at all (Fig. 6a, inset). The results with E_2_ATP plus 1,2-DCB started with Ca^2+^ at 0 or 25 °C were slightly different (Fig. 6b), and it appears that in this case the observed result could be more influenced by temperature. However, beside the temperature difference, a somewhat higher level of EP can still be observed at 0.05 mM of 1,2-DCB (Fig. 6b) when compared to the levels observed on Fig. 6a at the same DCB concentration, suggesting a possible protective effect by ATP, although experiments carried at zero degrees using DCB concentrations above 0.1 mM showed complete inhibition of EP formation (Fig. 6b, closed circles), but EP levels at 25 °C are greater than those observed at 0 °C. It is worth noting that the data shown in both figures does follow a similar trend, that is a consistent reduction of EP levels as the 1,2-DCB concentration increases.

## Discussion

Our data show that 1,2-DCB exerts a dual response of stimulation and inhibition in SERCA hydrolytic activity supported by A23187 (Fig. 1). Similar effects of activation and inhibition of SERCA caused by other hydrophobic compounds have been reported in the literature, and possible explanations include alterations in the lipid environment of the enzyme, as well as accessing sensitive domains of the pump due to their hydrophobic properties [49–51]. The 1,2-DCB molecule possesses two Cl atoms in the benzene ring. In order to test the importance of the Cl atoms on the effect of this molecule on SERCA, we used benzene (which lacks Cl atoms) and we were able to avoid the inhibitory and stimulatory effects on SERCA activity produced by 1,2-DCB (Fig. 1). Our data supports a clear relation between the structure of 1,2-DCB and the effect produced on the ATP hydrolytic activity, since the absence of Cl atoms does not cause any effect on SERCA. Similar conclusions have been previously reported regarding the effects caused by the presence of electronegative groups [52–54]. Moreover, hydrophobic molecules can interact with hydrophobic domains in the membrane proteins, thus affecting enzyme functionality [37, 53, 55].

We also addressed the possibility of 1,2-DCB affecting the activity of binding/translocation of Ca^2+^ by SERCA. Surprisingly, a 1 mM concentration of 1,2-DCB fully inhibited Ca^2+^ uptake without affecting Ca^2+^-binding, which suggests that the binding of Ca^2+^ is not a major problem for the inhibition of Ca^2+^ transport. However, this is indicative that 1,2-DCB is affecting the Ca^2+^ sensitive conformation of the enzyme to a lesser extent (Figs. 2a and 3 respectively). Nevertheless, our data of binding of Ca^2+^ suggested that Ca^2+^ was able to bind to the enzyme in turnover conditions almost at normal levels, even when Ca^2+^ uptake was completely inhibited at the highest DCB concentration. Therefore, it was necessary to study the hydrolytic activity of SERCA under the same conditions used for the Ca^2+^ transport assay, using K-oxalate instead of the Ca^2+^ ionophore. When intravesicular Ca^2+^ levels get higher (~2–3 mM), Ca^2+^ reacts with oxalate to form calcium oxalate thus precipitating inside of the intact vesicle as crystals [13], which allows us to measure the enzyme activity in min. Surprisingly, we found that the hydrolytic activity of SERCA under these same conditions was still stimulated, but not inhibited at the same concentrations tested before (Fig. 3b). This may be due to the different experimental conditions such as the presence of ionophore, which allows the system to function more efficiently. The Ca^2+^-ionophore acts as a freely mobile carrier that transports Ca^2+^ across the membrane, and it is possible that it is acting simultaneously with the 1,2-DCB, stimulating the pump due to increased membrane permeability and absence of gradient [13, 56, 57]. This could explain why activation and inhibition are more pronounced when measured with the Ca^2+^-ionophore than when measured with K-oxalate, where the vesicle interior is not gradient-free. We also found that the Ca^2+^/P_i_ ratio was partially uncoupled at 1,2-DCB concentrations between 0.05 and 0.5 mM, and fully uncoupled at 1 mM. This phenomenon of uncoupled Ca^2+^-ATPase with a lower Ca^2+^/P_i_ ratio has been previously described in terms of the increased thermogenic activity that occurs when using intact vesicles [13–15], where it was found that after Ca^2+^ saturation, ~82 % of the hydrolytic activity is uncoupled from the Ca^2+^ transport, thus decreasing the Ca^2+^/P_i_ ratio.

Previous reports showed that non-polar organic solvents facilitate the incorporation of hydrophobic compounds to cell membranes, and that enzymatic stimulation or inhibition is related to both solvent and membrane protein quantity [38, 49]. In agreement with the literature, 1,2-DCB affects SERCA activity by sharing these features, as we further demonstrated that the inhibitory effect of 1,2-DCB was SR membrane-dependent (Fig. 4). This could be due to a partitioning effect of the DCB into the membrane fraction, which indicates that activation or inhibition is dependent on membrane composition and protein concentration. Organic solvents can reduce the inhibition of SERCA by certain drugs, which explains how hydrophobic drugs can move to a lipid environment in the membrane, thus causing enzyme inhibition [49, 50, 58]. Data from Lax et al. [59] showed that the addition of phosphatidylcholine liposomes to SERCA reactions caused a decrease in the inhibitory effect of the fungicide miconazol. 1,2-DCB shares similar features to those of miconazole and therefore, we believe that our data indicates that DCB is going into the SR vesicles because of its hydrophobic properties.

The Ca^2+^ uptake assay employed in this study measured accumulated Ca^2+^ inside the SR vesicles [48, 60]. However, if 1,2-DCB disrupts the time required to fill the vesicles with Ca^2+^, this may be reflected as an inhibition on the accumulated Ca^2+^ due to a loss of Ca^2+^ caused by vesicle damage. Molecules such as 1,2-DCB can target SR membranes affecting the filling time of SR vesicles by the SERCA pump. For example, hexachlorocyclohexane, a hydrophobic compound, was shown to affect the activity of membrane proteins by disrupting the lipid bilayer [55]. For this reason, we filled out SR vesicles with Ca^2+^ using the active transport of SERCA, followed by the addition of a high concentration of DCB (1 mM) 5 s before stopping the reaction. In agreement with our previous findings, 1,2-DCB was unable to decrease the levels of accumulated Ca^2+^ when compared to controls (without DCB) (Fig. 5). Given that vesicle filling occurs in less than 1 s, which is the physiological time during which muscle relaxation occurs [15], we selected a longer vesicle filling time. This data clearly demonstrates that 1,2-DCB is not affecting the vesicle filling time by causing disturbances of the SR membrane. This was supported by our a positive control using Ca^2+^ ionophore A23187 (absence of gradient) alone or with 1 mM 1,2-DCB, which did not allow accumulation of Ca^2+^ inside the SR vesicles (Fig. 5). This phenomenon is referred to in the literature as “leaky vesicles”, since this system allows the efflux of luminal Ca^2+^, thus obtaining faster efflux [61, 62]. It was interesting to find that the SR membrane was not disrupted, which supports our findings that 1,2-DCB is causing uncoupling of the Ca^2+^/P_i_ ratio. Considering this phenomenon applied to a biological system, we can refer to the thermogenic activity previously proposed by Inesi and Tadini-Buoninsegni [15], where SERCA1 uncoupling interferes with the decrease of cytosolic Ca^2+^ and the subsequent relaxation of muscle fibers. If we take into account that vesicles are filled with Ca^2+^ in less than 1 s, a sufficient concentration of luminal Ca^2+^ would not be reached, thus preventing the dissociation of the E_2_-P^.^Ca_2_ form. This phenomenon could not lead to thermogenic activity due to a basal state of the pump. The Ca^2+^ leakage from the pump could occur if the luminal Ca^2+^ concentration reached such high levels that both its E_2_-P^.^Ca_2_ dissociation constant and the cytosolic Ca^2+^ concentrations would remain above the required levels to achieve maximal pump activation, which could occur under prolonged muscle activity. Even though the cytosolic Ca^2+^ levels can remain high due to multiple action potentials, this does not appear to be our case, since we have no evidence of pump overload (Figs. 2a and 5).

In order to get more insights into the mechanism by which 1,2-DCB caused uncoupling of the SERCA activity, we studied the E_1_P step of the enzyme. Our results demonstrated that 1,2-DCB decreased the EP stage formation but was influenced by temperature when starting from E_2_ATP, even when our previous experiments showed that 1,2-DCB produced a high rate of ATP hydrolysis. Our initial expectation was to observe elevated levels of EP by ATP, since it is common that hydrophobic molecules affect the E_2_ state of SERCA which prevents or decreases the transition to the E_1_ stage [25, 63]. Our data of EP inhibition suggests that 1,2-DCB (Fig. 6) could be affecting the E_2_ step. Similar examples of this phenomenon are found in the literature [15, 49, 51, 64, 65]. Taken together, the inhibition of the EP stage and our previous results could explain why an uncoupled Ca^2+^/P_i_ ratio was observed, even when Ca^2+^ is binding to the enzyme in the presence of 1,2-DCB. Phosphorylation of the enzyme is necessary for the appropriate Ca^2+^ transport across the SR membrane. Therefore, it is possible that a Ca^2+^ slippage process is taking place, where ATP hydrolysis is occurring without the subsequent accumulation of the transient E_1_P form and without Ca^2+^ transport, with Ca^2+^ “slipping” into the outside of the vesicles. This can be affected by several factors such as time, temperature, enzymatic stage and in our case the DCB concentration [66, 67] Our data shows that at 25 °C, EP levels were increased (Fig. 6b). However, the measurements followed the same pattern of EP decrease with increasing DCB concentration. Regarding the EP measurements conducted at 0 °C, which seem to go against the literature, it is well established that high levels of EP are obtained at low temperatures (0 °C) due to the rapid transition of the limiting E_1_P^.^Ca_2_ step to E_2_P^.^Ca_2_, and the subsequent Ca^2+^ translocation that leads to the EP decomposition [68], in other words, the phosphoenzyme formed from ATP is more sensitive to ADP at 0 °C than at 30 °C, thus confirming that ATP hydrolysis coupled to Ca^2+^ efflux and the ATP↔P_i_ exchange reaction are inhibited at 0 °C [69]. However, the experiments starting from E_2_ATP showed higher levels of accumulated EP, but these experiments were conducted at 0 and 25 °C, where A23187 is also present (although protein concentration, time and ATP are different). At 0.05 mM DCB, ~30–40 % of accumulated EP can be observed at both temperatures, indicating that ATP could be protecting the enzyme from inhibition. However, higher DCB concentrations (0.1 mM and subsequent) caused a drastic decrease in EP levels (Fig. 6b, filled circles). Other factors that could affect the E_1_P to E_2_P transition include detergents and the microviscosity of the lipid environment of the ATPase [70]. Temperature is another important factor that could potentially affect this transition, as it has been demonstrated that at 21 °C the E_1_P to E_2_P isomerization is rapidly affected at high KCl concentrations [71]. Temperature is also important for compound solubility in aqueous solutions, as it has been shown that the solubility of aroma compounds (such as methyl ketones, ethyl esters, aldehyde and alcohol) decreases when incubated on ice as the concentration of the compound is increased, which could contribute to explain the effect of temperature and DCB solubility at 0 and 25 °C in the EP measurements [72].

## Conclusions

Our data demonstrates that 1,2-DCB affects the ATP hydrolytic activity of SERCA, causing both stimulation and inhibition of ATP hydrolysis. This dual effect caused by 1,2-DCB fits it into a common pattern displayed by several other hydrophobic compounds. We also found that 1,2-DCB partially inhibited Ca^2+^ transport and EP stage formation due to uncoupled enzyme activity, even without affecting the Ca^2+^ bound to the enzyme. Finally, to the best of our knowledge, this is the first study designed to study the effect of 1,2-DCB, a chemical that is widely found in the environment, on the functionality of SERCA proteins, and thus the data presented here not only provide insights into the molecular mechanisms of the enzyme’s catalytic cycle, but on the possible toxic effects caused by 1,2-DCB.
